# Trends in Endoscopic and Open Hip Abductor Tendon Repairs: An Insurance Claims-Based Analysis from 2016 to 2022

**DOI:** 10.1007/s43465-026-01857-4

**Published:** 2026-06-26

**Authors:** Michael D. Greenstein, Genaro D. Fullano, Liam A. Peebles, Daniel G. Whitney, Matthew J. Hartwell

**Affiliations:** 1 University of Michigan Medical School, University of Michigan, Ann Arbor, MI USA; 2 Department of Orthopaedic Surgery, University of Michigan, Ann Arbor, USA; 3 Institute for Healthcare Policy and Innovation, University of Michigan, Ann Arbor, USA

**Keywords:** Gluteus medius, Gluteus minimus, Endoscopic repair, Open repair, Marketscan, Arthroscopy

## Abstract

**Purpose:**

To report national trends, including rates of open vs endoscopic, in hip abductor tendon repair surgery.

**Methods:**

This retrospective cohort study accessed patient-level insurance claims from adults ≥ 18 years undergoing endoscopic or open gluteal repair from 2016 to 2022 from the IBM^®^ MarketScan^®^ Commercial Database and a Medicare sample. The annual repair trends, stratified by insurance database, were described based on five categories: (1) all-cause; (2) proportion of repairs combined with hip arthroscopy or arthroplasty; (3) sex; (4) median age; (5) U.S. region of residence.

**Results:**

9217 total repairs across the study period were identified: 4504 in the Commercial and 4713 in the Medicare databases. In both datasets, > 50% of repairs occurred in women and the median patient age was > 50 years. The rate of endoscopic gluteal repairs declined from 2016 to 2022: Commercial (2.7 to 1.5 per 100,000 people) and Medicare (5.1 to 2.1 per 100,000 people). The rate of open gluteal repairs increased from 2016 to 2022: Commercial (0.4 to 1.0 per 100,000 people) and Medicare (1.0 to 2.5 per 100,000 people). The occurrence of gluteal repairs combined with arthroscopy or arthroplasty was rare and did not differ significantly between the two databases.

**Conclusions:**

Hip abductor tendon repairs were most performed in women and in patients over the age of 50. Across both the commercial and Medicare database, the rate of endoscopic tendon repairs decreased from 2016 to 2022, while open repairs increased. These descriptive trends provide insight into national practice patterns for gluteal tendon tear repairs.

## Introduction

The gluteus medius and minimus (hip abductor) tendons are essential for hip joint stability and gait mechanics [1, 2]. Tears of the hip abductor tendons have been increasingly recognized as a source of pain in greater trochanteric pain syndrome (GTPS) [3]. Improved recognition and diagnostic imaging [4, 5] has led to the identification and repair of these tendon injuries becoming a recent focus of orthopaedic investigation [6–8].

Morgan et al. in 2024 conducted a review of surgical treatment options and outcomes for gluteal tendon tears outlining that operative management involves repair vs. reconstruction vs. tendon transfer and that these may be completed through endoscopic vs. open techniques [9]. The open approach to gluteal tendon repair was the historical gold standard and may still be preferentially employed in the repair of large, full-thickness, or retracted gluteal tears [6, 10]. Recent studies have evaluated outcomes following endoscopic repair [11, 12] and comparisons between endoscopic and open approaches have yielded similar functional and patient-reported outcomes [6, 7, 13].

GTPS itself is common, particularly among middle-aged female patients [14], with an estimated incidence of 1.8 per 1000 patients per year [15]. However, the actual rate of GTPS due to hip abductor tendon injury and the yearly operative trend are unknown. Despite increasing anecdotal and literature reports on gluteal tendon repairs, there is an apparent lack of peer-reviewed investigations into national repair trends for gluteal tendon tears. Health claims databases have previously been employed to investigate such surgical trends in hip surgery [16, 17] and may be an important tool in investigating this gap in gluteal tendon repair trends across the United States (U.S). In particular, the Marketscan Databases from Truven Health Analytics are known for their yearly continuity of included subjects, making them a strong candidate for an operative trends analysis [18].

The primary aim of this investigation was to determine the year-over-year trend of gluteus medius and minimus endoscopic vs. open tendon repairs using commercially available healthcare databases. The secondary aims of this investigation were to determine the year-over-year trend of gluteal tendon repairs stratified by isolated vs. combined (arthroscopy and arthroplasty) cases, sex, age, and U.S. region of residence.

## Methods

### Data Source

This was a retrospective cohort study that accessed claims data from January 1, 2016, through December 31, 2022, from two separate administrative claims databases, including the Commercial Database from the IBM^®^ Watson Health MarketScan^®^ Research Databases and a 20% random sample of the Medicare fee-for-service database (Part A and B). These databases were included, because the Commercial database is highly representative of the general working, non-elderly population < 65 years of age, while the Medicare database is highly representative of the elderly population ≥ 65 years of age [19]. Including both cohorts captures a large, heterogeneous cohort across the adult lifespan with geographic diversity, thus enhancing representation and generalizability of study findings.

The IBM^®^ MarketScan^®^ Commercial Database includes health insurance-related data from the private-sector encompassing ~ 350 payers across the U.S. and represents individuals that are insured employees, continuees of the Consolidated Omnibus Budget Reconciliation Act, and non-Medicare eligible dependents that have insurance coverage based on employer-sponsored plans. Medicare is a federal program providing health insurance to the elderly, as well as elderly and non-elderly with specific disabilities or with end-stage renal disease. Medicare beneficiaries can have dual eligibility with Medicaid, but for those with dual-eligibility, Medicare pays first for services that are covered by Medicare and Medicaid, including the services needed to conduct this study. All claims data are de-identified prior to administering to researchers. Therefore, patient consent was not required. As a result, the Institutional Review Board was obtained and this study was deemed as exempt and “Not Regulated”.

### Gluteal Repair

To conduct research studies using data derived from administrative claims, surgical procedures (e.g., open/endoscopic gluteal repair) are identified by Current Procedural Terminology (CPT) codes. The CPT coding algorithm for gluteal tendon repairs does not rely on a single CPT code. The standard of practice presently involves using “unlisted” codes in combination with reference codes to bill for gluteal tendon repairs. Our search followed the same algorithm. Procedures were identified by using the unlisted procedure codes for the endoscopic (CPT code: 29,999) and open (CPT code: 27,299) approach combined with a same day reference code for endoscopic (CPT code: 29,827) and open (CPT codes: 23,412, 23,420, 27,385) rotator cuff repair surgery. This approach does not delineate full-thickness and partial-thickness gluteal tendon tears. Partial-thickness tears, managed with debridement instead of full repair (and subsequently were coded through a different CPT mechanism), were not included in this analysis.

Gluteal tendon tears can present with concomitant intra-articular pathologies [20]. The choice of endoscopic vs. open approach may be influenced by a concomitant injury. To account for this, we additionally determined how many of the endoscopic and open gluteal repairs had concomitant hip arthroscopy (CPT codes: 29,860, 29,861, 29,862, 29,863, 29,914–29,916) and hip arthroplasty (CPT codes: 27,130, 27,132, 27,134, 27,033) surgery on the same day.

### Descriptive Characteristics

Year of the surgery, age at surgery, sex, and U.S. region of residence were retrieved from the databases. The Medicare database provides the date of birth (DD/MM/YYYY) allowing for a precise age calculation, while the Commercial database provides the year of birth (YYYY) leading to a less-precise age calculation. The Commercial database does not provide information about race or ethnicity, and as such, these variables were not assessed in this study. To maintain descriptive focus strictly on yearly operative trends, medical comorbidities were similarly not assessed.

### Statistical Analysis

To assess annual trends, the primary outcome was the number of surgeries per calendar year from 2016 through 2022. Participants could contribute to multiple years so long as they had distinct, eligible surgeries in different calendar years. For participants that contributed to multiple years, each surgery had corresponding data relevant to that year of surgery (e.g., age was updated). To calculate the annual rates, the denominator included all participants ≥ 18 years old (age calculated based on January 1st of each calendar year) that did not receive the surgeries of interest, per calendar year, from each database irrespective of their length of enrollment. The annual rate was calculated as the number of surgeries divided by the denominator per calendar year multiplied by 100,000.

The analytic plan was purely descriptive, and all analyses were stratified by the insurance database given the differences in the vastness (e.g., 20% sample of Medicare; full sample of Commercial), enrollment criteria (e.g., working, elderly, or non-elderly but with certain qualifying disabilities), and representation. The insurance database-stratified annual trends in endoscopic and open gluteal repairs were described in five ways: first, by all-cause (i.e., isolated, arthroscopy, arthroplasty); second, by the proportion based on isolated or in combination with either arthroscopy or arthroplasty; third, by sex; fourth, by the median age; and fifth, by U.S. region of residence.

Any stratification resulting in n ≤ 11 patients was suppressed according to the Data Use Agreements (DUA) to maintain HIPAA-compliant de-identification. Analyses were performed using SAS version 9.4 (SAS Institute, Cary, NC, USA).

## Results

There were 9217 total repairs across the study period: 4504 in the Commercial and 4713 in the Medicare databases. The majority of procedures were performed on women in both the Commercial (52.6%) and the Medicare (62.9%) databases. The median (IQR) patient age was 57 (51–61) in the Commercial database and 70.2 (67.0–74.5) in the Medicare database. Full descriptive characteristics are presented in Table 1.

**Table 1:** Combined demographic characteristics for endoscopic or open gluteal hip repairs from 2016 to 2022

	Commercial (*n* = 4504)		Medicare (*n* = 4713)	
	Median	IQR	Median	IQR
Age in years	57	51.0, 61.0	70.2	67.0, 74.5
	*N*	*%*	*N*	*%*
Sex				
Male	2136	47.4	1747	37.1
Female	2368	52.6	2966	62.9
U.S. region of residence				
Northeast	637	14.1	775	16.4
Midwest	1277	28.4	1314	27.9
South	1904	42.3	1699	36.1
West	664	14.7	925	19.6
Missing	22	0.5	0	0
Year of gluteal repair, endoscopic or open, surgery				
2016	858	19.1	730	15.5
2017	688	15.3	691	14.7
2018	709	15.7	673	14.3
2019	617	13.7	714	15.2
2020	532	11.8	640	13.6
2021	540	12.0	619	13.1
2022	560	12.4	646	13.7

**Fig. 1:**
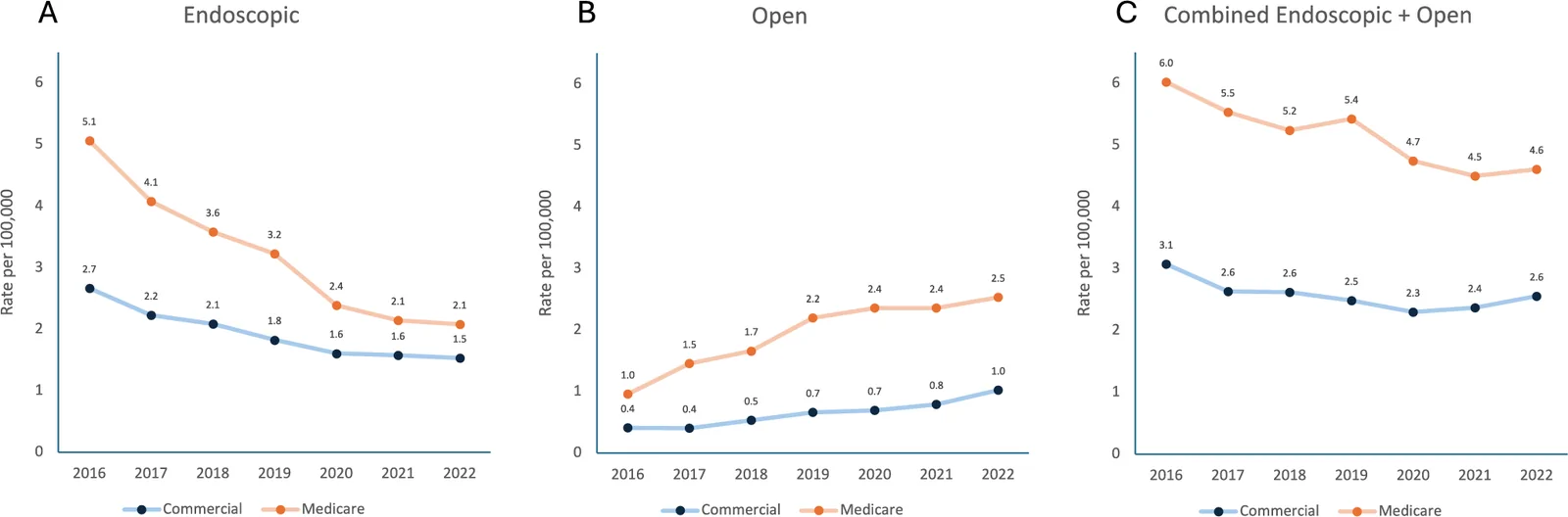
Rate of all-cause gluteal repair and year stratified by approach and insurance database. Legend: Year-over-year prevalence per 100,000 people all-cause gluteal repairs stratified by approach: endoscopic (**A**), open (**B**), and combined (**C**)

The primary outcome was the rate of all-cause gluteal repairs stratified by operative approach and database, as presented in Fig. 1. From 2016 through 2022, the rate of endoscopic repairs declined year over year from 2.7 per 100,000 patients to 1.5 per 100,000 patients (Commercial) and 5.1 per 100,000 patients to 2.1 per 100,000 patients (Medicare). Over the same study period, the rate of open repairs increased year over year from 0.4 per 100,000 patients to 1.0 per 100,000 patients (Commercial) and 1.0 per 100,000 patients to 2.5 per 100,000 patients (Medicare).

The secondary outcome was the proportion of gluteal repairs with a concomitant procedure (arthroscopy or arthroplasty), stratified by approach and insurance database, as presented in Table 2. No endoscopic procedures were combined with arthroplasty. In the Commercial database, the total number of endoscopic procedures combined with arthroscopy was less than 11 in 2016, 2017, 2018, and 2022. There were zero endoscopic repairs combined with arthroscopy in 2019, 2020, or 2021. In the Medicare database, all endoscopic repairs were performed in isolation. Open repairs were more frequently performed with concomitant arthroscopy or arthroplasty procedures which were combined into a single quantitative measure for this analysis due to MarketScan’s DUA.

**Table 2:** Proportion of gluteal repairs performed with concomitant arthroscopy or arthroplasty

Year of gluteal repair	Endoscopic		Open	
	*N* (%) Isolated	*N* (%) Combined ^*a*^	*N* (%) Isolated	*N* (%) Combined
Commercial				
2016	*N* (%) > 1^b^	*N* (%) > 1^b^	99 (86%)	16 (14%)
2017	*N* (%) > 1^b^	*N* (%) > 1^b^	92 (87%)	14 (13%)
2018	*N* (%) > 1^b^	*N* (%) > 1^b^	116 (80%)	29 (20%)
2019	453 (100%)	0 (0%)	135 (82%)	29 (18%)
2020	371 (100%)	0 (0%)	137 (85%)	24 (15%)
2021	360 (100%)	0 (0%)	156 (87%)	24 (13%)
2022	*N* (%) > 1^b^	*N* (%) > 1^b^	184 (82%)	41 (18%)
Medicare				
2016	614 (100%)	0 (0%)	87 (75%)	29 (25%)
2017	509 (100%)	0 (0%)	127 (70%)	55 (30%)
2018	460 (100%)	0 (0%)	169 (80%)	44 (20%)
2019	425 (100%)	0 (0%)	218 (75%)	71 (25%)
2020	322 (100%)	0 (0%)	253 (80%)	65 (20%)
2021	295 (100%)	0 (0%)	244 (75%)	80 (25%)
2022	291(100%)	0 (0%)	280 (79%)	75 (21%)

^a^Endoscopic procedures were never combined with arthroplasty, just arthroscopy

^b^Suppressed data based on MarketScan DUA

**Fig. 2:**
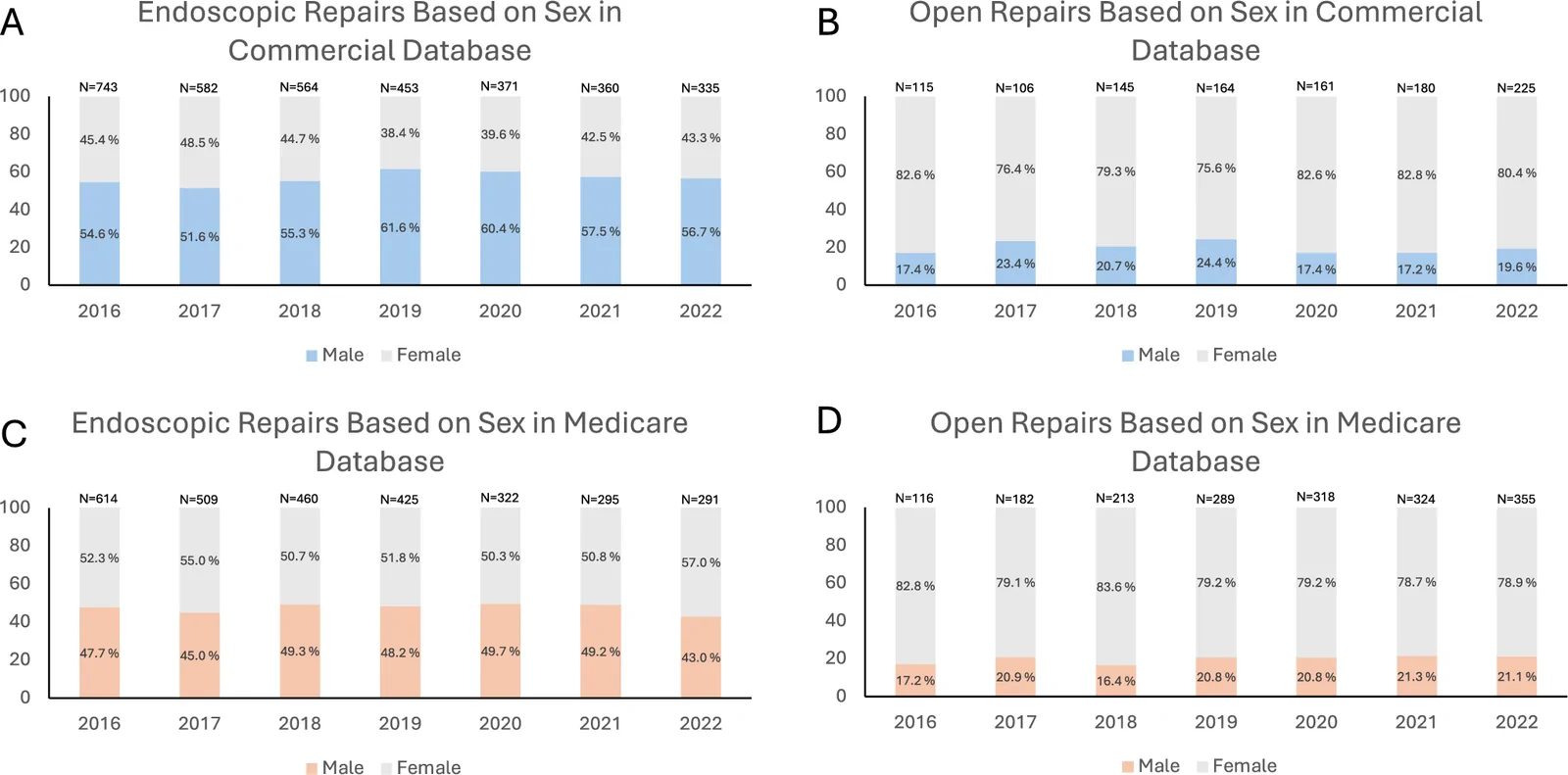
Proportion of all-cause gluteal endoscopic and open repairs stratified by sex and insurance database. Legend: Proportion of all-cause gluteal repairs stratified by sex for the Commercial database endoscopic (**A**), Commercial database open (**B**), Medicare database endoscopic (**C**), and Medicare database open (**D**). Individual data values indicate sex-based percentages per year

Sex stratification revealed that most gluteal repairs were performed on female patients in both the Commercial (52.6%) and Medicare (62.9%) databases Table 1. The year-over-year proportion of male-to-female gluteal repairs stratified by approach and database is presented in Fig. 2. Regardless of database, the endoscopic approach revealed a more balanced percentage split by sex with female procedures ranging from 38.4 to 57.0% compared to the open approach which had female predominance: proportion of female procedures per year ranging from 75.6 to 83.6%.

**Fig. 3:**
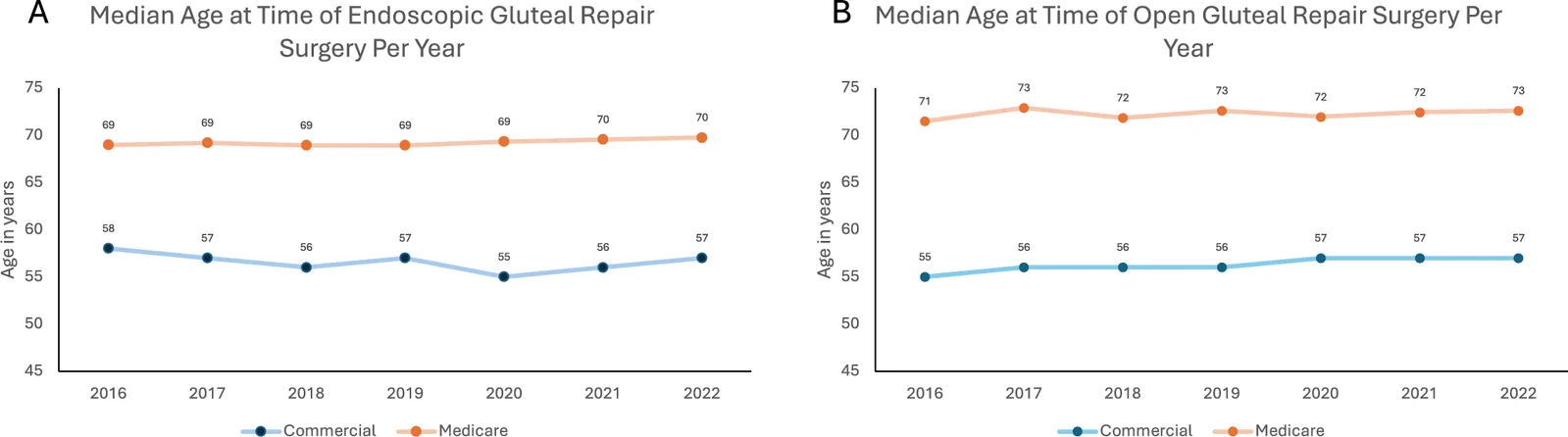
Median age at time of surgery per year stratified by insurance database and surgical approach. Legend: Median age at time of surgery, per year, stratified by database for the endoscopic approach (**A**) and open approach (**B**)

Median age at time of surgery per year was stable across each database, regardless of operative approach as presented in Fig. 3. The Commercial database had a median age range of 55–57 years for both endoscopic and open procedures. The Medicare database had a median age range of 69–70 years for endoscopic procedures and 71–73 years for open procedures. Endoscopic and open gluteal repairs stratified by U.S. region of residence are presented in Table 3 for the Commercial database and Table 4 for the Medicare database. Across both databases and operative approaches, the greatest proportion of procedures were performed in the South and Midwest regions.

**Table 3:** Proportion of gluteal repairs stratified by U.S. region of residence in the commercial database

Year of gluteal repair	Endoscopic				
	Northeast	Midwest	South	West	Total
	%	%	%	%	*N*
2016	13.2%	26.3%	48.2%	12.3%	741
2017	15.9%	22.5%	45.8%	15.7%	578
2018	17.2%	27.2%	39.9%	15.7%	559
2019	18.3%	29.6%	37.6%	14.5%	44
2020	13.3%	31.0%	40.8%	14.9%	368
2021	13.1%	34.8%	35.1%	17.0%	359
2022	13.7%	27.5%	43.9%	14.9%	335
Open					
2016	N > 1%^a^	N > 1%^a^	N > 1%^a^	N > 1%^a^	113
2017	14.2%	28.3%	40.6%	17.0%	106
2018	15.2%	31.7%	40.0%	13.1%	145
2019	14.6%	25.6%	43.3%	16.5%	164
2020	10.6%	34.2%	42.2%	13.0%	161
2021	10.0%	29.4%	46.1%	14.4%	180
2022	10.7%	32.1%	41.5%	15.6%	224

These data are missing procedures that were classified as “Missing” U.S. region of residence. Percentages per region are based on tabular *N* values

^a^Suppressed data based on MarketScan DUA

**Table 4:** Proportion of gluteal repairs stratified by U.S. region of residence in the Medicare database

Year of gluteal repair	Endoscopic				
	Northeast	Midwest	South	West	Total
	*%*	*%*	*%*	*%*	*N*
2016	17.6%	25.7%	40.4%	16.3%	614
2017	19.4%	24.6%	39.3%	16.7%	509
2018	18.9%	26.3%	35.0%	19.8%	460
2019	15.1%	25.9%	37.4%	21.6%	425
2020	16.1%	29.2%	35.4%	19.3%	322
2021	12.2%	26.1%	34.2%	27.5%	295
2022	15.1%	24.1%	34.4%	26.5%	291
	Open				
2016	12.9%	32.8%	33.6%	20.7%	116
2017	15.4%	29.7%	34.1%	20.9%	182
2018	15.5%	30.5%	34.3%	19.7%	213
2019	13.8%	35.3%	36.0%	14.9%	289
2020	19.5%	29.2%	34.3%	17.0%	318
2021	15.1%	29.9%	34.9%	20.1%	324
2022	16.3%	31.0%	32.7%	20.0%	355

## Discussion

The most important finding from the present study was that across both databases, endoscopic repairs demonstrated a year-over-year stagnation or decline, while open repairs increased from 2016 through 2022. When combining the operative approaches into a single variable, the overall trend demonstrated a marginal decrease in operative rate in the study window. Secondarily, open repairs were more commonly combined with concomitant arthroscopy or arthroplasty compared to the endoscopic approach. The majority of gluteal repairs occurred in women, the median patient age was older than 50 years old, and most procedures occurred the Southern U.S region.

There has been growing clinical evidence surrounding the use of the endoscopic approach compared to the open technique [6–8, 13]. In 2015, Chandrasekaran et al. conducted a systematic review of seven studies and 127 patients to compare functional and patient-reported outcomes in endoscopic vs. gluteal tendon repairs, and concluded that there was no significant difference between the two groups, but open repairs were associated with increased complication rates [7]. However, the authors note that the included endoscopic studies did not report any complication rates. Longstaffe et al. reported similarly in their 2020 systematic review of 22 studies and 611 hips finding comparable functional and clinical improvement between the endoscopic and open approaches with fewer complications in the endoscopic approach [13]. In 2021, Flynn et al. conducted a comparative study evaluating the biomechanical outcomes between endoscopic and open double-row gluteus medius repairs using a cadaveric model [8]. The investigators found that there was no significant differences in the ultimate load or gap formation between the two approaches. Moreover, Browning et al. in 2025 evaluated 2-year minimum outcomes for hip abductor tendon repairs stratified by imaging and surgical approach in 112 patients and concluded that both approaches showed significant improvements in patient-reported outcomes [6].

Despite the growing evidence supporting the adoption of the endoscopic technique, data collected within the current study showed a year-over-year rise in the utility of the open approach compared to a downtrending endoscopic approach.

The open approach has reported advantages over the endoscopic approach and this trend may reflect preference for these benefits. The open technique is often more familiar to surgeons and provides greater visualization [9] and has been reported to confer operative time advantages [21]. Open repair also remains the standard approach for large or retracted gluteal tendon tears and provides flexibility to convert to flap transfer [10]. As demonstrated in our reporting, the open approach can also more readily be combined with a concomitant procedure such as total hip arthroplasty, increasing its versatility. Prior investigations of combined open gluteal repair and arthroplasty have demonstrated positive outcomes. Maldonado et al. in 2021 reported good patient-reported outcomes on 30 hips with an average of 2-year follow up that underwent combined gluteal tendon repair through an open approach and primary total hip arthroplasty (THA) [22]. Similarly, Gaillard-Campbell & Gross in 2025 compared clinical hip arthroplasty outcomes (Harris Hip Score and Visual Analog Scale) between patients that had concomitant gluteal tendon repair and those that did not [23]. The researchers concluded combined arthroplasty and tendon repair does not alter functional restoration. The endoscopic approach may be gaining academic interest, but the identified trend within the current work may reflect a prioritization of the advantages and comfort with the open repair approach.

Selecting the familiar operative approach is not a new phenomenon as orthopaedic surgeons tend to prefer techniques they were initially taught [24, 25]. Grove et al. expand on this idea in a 2018 investigation into the role of evidence and knowledge in orthopaedic decision-making [24]. The authors describe the complex and nuanced process by which decision-making is conducted in orthopedics and how experience and training interact with knowledge in evidence-based medicine. Scantlebury et al. further identify the potential discordance in orthopaedic decision-making between evidence and experience, with experience often being favored [26]. Real-world support for preference of experience over newer evidence may be gleaned from practice patterns for orthopaedic surgeries, specifically shoulder and knee arthroscopy, which have been shown to vary across different U.S. regions despite centralized data and reports of procedure effectiveness [27]. These variations may exemplify the difference in incorporation of new evidence into actual practice.

Given the relative commonness of GTPS, the total number of gluteal repairs in the study window was surprisingly low. This may be a consequence of the opaque coding process used in the CPT billing for gluteal tendon repairs. The coding for gluteal tendon repairs is circumferential and relies on combining unlisted and reference codes. Without a direct coding pathway, it is difficult to ascertain the criterion validity of using the two-step CPT code search to identify gluteal tendon repairs. Thus, it is possible that we captured only a subset of the gluteal tendon repairs being performed in the MarketScan Databases. Alternatively, we may have accurately captured the entirety of procedures being performed, but the Databases themselves are limited in the true sampling of these injuries [18]. Moreover, the two-step CPT code search may preferentially have favored full-thickness tears over partial tears. To identify endoscopic gluteal tendon repairs, the unlisted CPT code 29,999 was used in conjunction with the reference CPT code 29,827 (“arthroscopy, shoulder, surgical; with rotator cuff repair”). This reference code, while commonly used, relies on operative documentation demonstrating similarity to the reference code which is commonly a full-thickness repair. As such, there may be a portion of endoscopically repaired partial-thickness tears that are coded through a different hip arthroscopy CPT code. It is challenging to confirm if the observed trend is more strongly a consequence of practice patterns or due to a bias in the data query.

There are a few key strengths in the presented approach supporting the external validity of this analysis. When conducting the initial data query, input from surgeons at our own institution who commonly perform this procedure was solicited and a review of the literature was completed to identify the most common coding algorithms for these tendon tears [28, 29].

Additionally, most procedures were performed on women and the median age was greater than 50 years which is the expected of gluteal tendon repairs. Moreover, the data query and interpretation were overseen by a trained data analyst with significant experience conducting large database research. This helped reduce the risk of overinterpretation of the findings due to lack of understanding of the database itself [30].

The use of the MarketScan Commercial Databases for trend analysis is not without limitations. The assessment of comorbidities was not included, and thus, the trend results are purely descriptive without mention of health factors affecting rates of operation. The Commercial Database does not include race and ethnicity, and therefore, these were not included in the analysis. The Commercial and Medicare Databases are sampled differently and represent two distinct populations in the United States, restricting our ability to combine data from these two Databases.

## Conclusion

Hip abductor tendon repairs were most performed in women and in patients over the age of 50. Across both the commercial and Medicare database, the rate of endoscopic tendon repairs decreased from 2016 to 2022, while open repairs increased. Open repairs were more commonly associated with concomitant procedures. These descriptive trends provide insight into national practice patterns for gluteal tendon tear repairs.

## Declarations

### Image publication

All images included in this study are original and have not been published before.
